# Heuristic Analysis Model of Nitrided Layers’ Formation Consisting of the Image Processing and Analysis and Elements of Artificial Intelligence

**DOI:** 10.3390/ma9040265

**Published:** 2016-04-01

**Authors:** Tomasz Wójcicki, Michał Nowicki

**Affiliations:** 1 Institute for Sustainable Technologies, National Research Institute, Pulaski Str. 6/10, 26-600 Radom, Poland; 2 Industrial Research Institute for Automation and Measurements PIAP, Jerozolimskie 202, 02-486 Warsaw, Poland; nowicki@mchtr.pw.edu.pl

**Keywords:** image analysis, surface layer, gas nitriding, artificial intelligence

## Abstract

The article presents a selected area of research and development concerning the methods of material analysis based on the automatic image recognition of the investigated metallographic sections. The objectives of the analyses of the materials for gas nitriding technology are described. The methods of the preparation of nitrided layers, the steps of the process and the construction and operation of devices for gas nitriding are given. We discuss the possibility of using the methods of digital images processing in the analysis of the materials, as well as their essential task groups: improving the quality of the images, segmentation, morphological transformations and image recognition. The developed analysis model of the nitrided layers formation, covering image processing and analysis techniques, as well as selected methods of artificial intelligence are presented. The model is divided into stages, which are formalized in order to better reproduce their actions. The validation of the presented method is performed. The advantages and limitations of the developed solution, as well as the possibilities of its practical use, are listed.

## 1. Introduction

Gas nitriding is a thermo-chemical treatment of iron alloys involving the diffusion saturation of the metal surface with nitrogen. The aim of the thermo-chemical treatment is to produce structural changes in the processed material and, thus, to change its functional characteristics and mechanical or physico-chemical properties (*i.e.*, abrasion resistance, hardness, fatigue resistance, corrosion resistance). The process includes several steps: the production of the active saturating elements’ atoms, forming a nitriding atmosphere; the adsorption of free atoms on the surface of the workpiece, including physical adsorption associated with the attraction of atoms and chemical adsorption associated with the formation of the intermetallic layer; thermally-activated diffusion of atoms in the surface layer involving the spread of the molecules of the diffusing substances in the surrounding medium. During the nitriding process, the nitrogen concentration in the surface layer is changed. Diffusing nitrogen creates a zone of chemical compounds composed of pure nitrides and a diffusion zone (internal nitriding zone), wherein the content of nitrides is reduced to the negligible value in the core material. The whole process can be controlled, among other things, by creating the nitriding atmosphere with a certain potential, produced by different techniques and providing it with a specified flow through the retort. In the case of the bicomponent atmosphere, the dilution of ammonia (NH_3_) with nitrogen (N_2_) or dissociated ammonia (NH_3_dis) results in the reduction of the supply of atomic nitrogen on the steel surface, which leads to a reduction of the surface concentration of nitrogen and, consequently, to limiting the growth of undesirable, porous and brittle ε, ε + γ' zones. The effect of the nitriding process is the outer layer of the structure and the phase composition dependent on parameters, such as the temperature, the duration of the process, the type of material constituting the object subjected to nitriding and the nitriding atmosphere composition.

**Figure 1:**
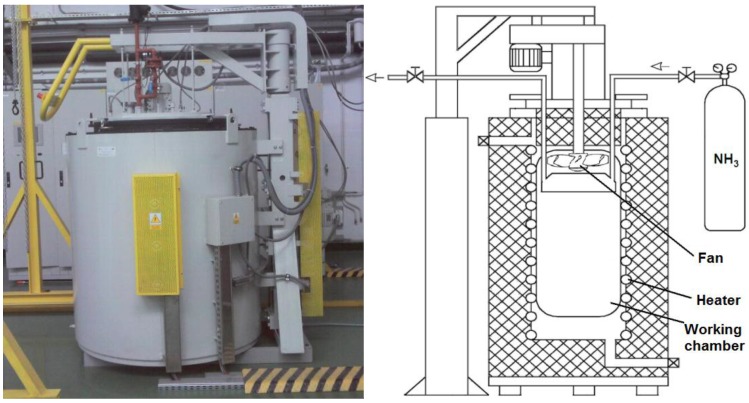
Photograph and a schematic diagram of the gas nitriding device.

Nitriding is done on materials comprising Cr, V or Mo nitride-creating elements. During the nitriding process, such phases are formed as nitric ferrite, nitric austenite, γ' nitrides (Fe_4_N) and ε nitrides (Fe_2_N). The gas nitriding process is carried out in specially-designed machines, which, depending on the type of gas nitriding, may vary the equipment. The typical facility to carry out gas nitriding (Figure 1) consists of: retort with a system of heaters, a dissociator, inlet and outlet gas systems, the fan circulating the atmosphere and the control system. In addition, the machine can be equipped with sensors to allow the control of the processes parameters, including determining the degree of ammonia dissociation and nitrogen potential, and the sensors allowing for the investigation of the process results (e.g., magnetic field sensor [1]).

Thermo-chemical treatment in the form of gas nitriding is utilized in a number of engine and pump components’ production. The processed parts are, e.g., connecting rods, cylinder liners, pistons, pins and piston rings, gears and shafts [2]. Nitriding is also widely used to improve the tools’ durability, such as tools for plastic forming, forging dies, molds, drawing dies, cutting tools made of high speed steel, drills, taps, milling cutters, *etc.*

## 2. Methods of Processing and Analysis of Digital Images

The processing and analysis of digital images is the area of problems related to the processing and interpretation of video signals using information technology. Automatic processing and analysis of digital images is widely used in cognitive [3] and practical applications and is utilized in many industry sectors [4]. There are many types of digital image processing and analyzing methods, and their essential task groups are: improving the quality of the images, segmentation, morphological transformations and image recognition. Methods for improving image quality [5] are used to reduce interference associated with non-linear characteristics of optoelectronic transmitters or aberration of lens systems used in vision systems.

The methods of improving image quality include point (anamorphic) and spatial operations. Point operations are characteristic in that the result of the conversion of pixels depends only on the values of the analyzed pixel, without taking into account the relationship with other pixels located in their vicinity. In the case of spatial operations, analyzed pixel values depend on the values of other pixels from a specific neighborhood or environment dependent on the diameter of the so-called local operator. Another class of methods for processing and analyzing digital images is the segmentation process, also known as labelling [6]. The segmentation process operations involve the division of data forming the image in order to increase the efficiency of recognition and the interpretation of the objects therein. Segmentation methods include two basic groups: area and contour. Area segmentation is a division of the image into homogeneous zones, meeting the established criteria of homogeneity (e.g., intensity, color, texture), which typically represent specific fragments of reality mapped to the picture. Contour segmentation is used to determine the boundaries between objects mapped in the analyzed image. Examples of methods of segmentation are: thresholding [7], region-growing [8] and watershed transformation [9]. Another group of methods for image processing and analysis are morphological transformations, by means of which the processing of objects’ shapes, which are mapped in the analyzed image, is carried out. This is done through the use of so-called structurizing elements [10], which are patterns of specific shapes with highlighted center points. Because of the complexity of morphological transformation algorithms, they are divided into: simple (primary) transformations and complex transformations. Simple transformations are characterized by the fact that they cannot be replaced with other morphological transformations. Complex transformations are divided into the first order and the transformations of the second or higher order. The first order complex transformations are characterized by the fact that their action is based on a few basic operations, and the number of occurrences of different types of basic operations is limited to one. Complex transformations of the second and higher order are transformations whose operation is based on a combination of basic operations; the number of occurrences of each basic operations for the second order is two and for higher order, proportionately more. An important set of methods for image processing and analysis are pattern recognition algorithms. There are many definitions of image recognition process. Image recognition by Duda and Hart is a machine identification of significant regularities in a complex and noisy environment [11]. Bezdek defines pattern recognition as a search for graphical data structures [12], and Tadeusiewicz defines the image recognition as automatic identification of classes, namely recognition of objects and phenomena belonging to the classes [13]. The image recognition process usually consists of two stages. The first stage is a measurement of the value of the characteristic features of the objects recognized in the image.

**Figure 2:**
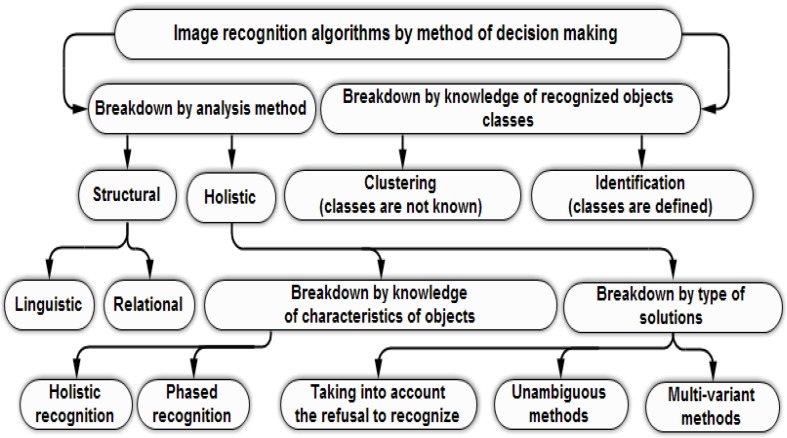
Classification of image recognition algorithms due to the implementation of the decision making.

The second step provides the analysis of the measured feature values to determine the membership of objects characterized by the features to defined classes. Features, by which objects are characterized, may be different for different types of objects; thus, it is not possible to unify them. Pattern recognition methods, divided due to the implementation of the decision making, are shown in Figure 2.

The choice of methods for image processing and analysis and their use depends on many factors, among which a significant role is played by the type of objects represented in the analyzed images and the assumed limitations of processing time. Different kinds of methods are characterized by specific sets of advantages and limitations, determining their practical application. The effective selection of methods to solve the given problem determines to a large extent the achieved results.

## 3. Model of the Processes of the Manufacture of Nitrided Layers’ Analysis Based on Automated Analysis of Digital Images

**Figure 3:**
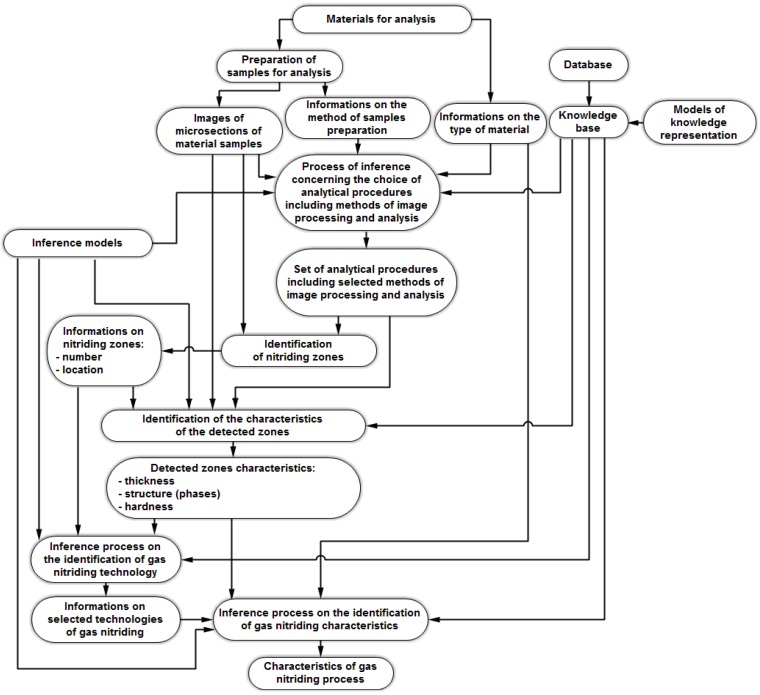
The structural model for studying the preparation mechanisms of nitrided layers based on automated analysis of digital images.

The developed solution makes it possible to identify the conditions under which the chosen coatings were manufactured, based on a limited range of input data in the form of digital image. The tasks of this type fall within the area referred to as reverse engineering and are used *inter alia* for the purpose of discovering the method used to develop certain products. As for the practical aspects, it can be applied in the analysis of the causes of damage to machines and equipment or the investigation of the technology used by competitors. In the developed model, it was assumed that the automatic inference concerning the parameters characterizing the gas nitriding processes and the obtained layers’ properties will be based on metallographic section photos. The structural model of the method for studying the mechanisms of preparing nitrided layers based on automated analysis of digital images is shown in Figure 3.

The general model of formal analysis is as follows:

(1) $$F_{M}:X_{M}\to Y_{M}$$

where:

*F_M_*—the operator of the model, *X_M_*—input values space, *Y_M_*—output values space.

The input values space *X_M_* is composed of: metallographic sections’ digital images, information on the actual size of the mapped structures, information on the method of metallographic sections’ preparation and information about the type of material constituting the core of the analyzed sample. The output values space *Y_M_* is the layers’ parameters, information on the possible technologies used in gas nitriding and the process characteristics with which it is possible to produce surface layers similar to those that have been analyzed.

Before examining the images and if multi-stage inference is possible, it is necessary to properly prepare input data in the form of digital images of material samples. Photos should be so arranged that they exhibit the characteristics of the analyzed material structures in the best possible way. The metallographic sections should be prepared using techniques that allow visualization of their characteristics, such as etching, evaporation or coloring [14]. Reagents for etching effect primarily the grain boundaries, and grains of different composition are more or less etched. When the etched sample consists of several phases, then they are shown in different degrees, which allows carrying out the automatic identification based on the photos. It is important that the same analyzed structures are prepared by identical methods. Taking pictures of the material samples is possible with the use of metallographic microscopes currently available on the market, with the function of recording images in digital form, which ensures the repeatability of the obtained images. It is also important that the photos are made for the same sample orientation (e.g., the material surface cross-section is on the left side and the core on the right side of the photo). It is essential for further analysis of images, as analytical algorithms require that one specify the direction of analysis (the order of nitriding zones’ occurrence on the pictures).

The main analysis begins with the inference concerning the choice of procedures covering the methods of processing and analyzing digital images to identify nitriding areas and their characteristics. It is necessary in order to obtain the characteristics of the distribution of the brightness of pixels in the images representing metallographic cross-sections. A method of forming these characteristics shows, *inter alia*, Equation (3). This step is necessary because of differences related to the representation of the nitriding areas, resulting, *inter* *alia*, from the methods of sample preparation and the core material, which could affect the obtained picture.

Formally, this stage is as follows:

$$F_{S1}:X_{S1}\to Y_{S1}$$

where:

$X_{S1}=\{X^{P},X^{R},X^{Z}\}$—input values space for the inference associated with the identification procedures for image processing and analysis,
$X^{P}=\{x_{1}^{P},x_{2}^{P},...,x_{i}^{P}\}$—a set of parameters of metallographic section preparation,
$X^{R}=\{x_{1}^{R},x_{2}^{R},...,x_{j}^{R}\}$—a set of parameters characterizing the core material,
$X^{Z}=\{x_{1}^{Z},x_{2}^{Z},...,x_{k}^{Z}\}$—a set of parameters characterizing digital photos,
$Y_{S1}=\{Y^{U}\}$—output values section for the inference associated with identification procedures for image processing and analysis,
$Y^{U}=\{y_{1}^{u},y_{2}^{u},...,y_{l}^{u}\}$—a set of parameters characterizing the methods of processing and analyzing digital images included in the procedures for identifying gas nitriding areas.

The records are presented in the generalized (model) form. In individual instances, the parameters in different sets can have different values, e.g.:

Metallographic section preparation parameters:

$x_{1}^{P}$—6—sample preparation method identifier (combination of processes, for which a number of other parameters can vary),
$x_{2}^{P}$—0.63—the upper limit of the Ra roughness after the mechanical grinding process,
$x_{3}^{P}$—0.54—the lower limit of the Ra roughness after the mechanical grinding process,
$x_{4}^{P}$—0.02—the upper limit of the Ra roughness after the mechanical polishing process,
$x_{5}^{P}$—0.012—the lower limit of the Ra roughness after the mechanical polishing process,
$x_{6}^{P}$—4—etching method identifier (e.g., 4 is for Nital).

Core material parameters:

$x_{1}^{R}$—AISI Type H13 hot work tool steel—material identifier (it indicates a material having a particular chemical composition),
$x_{2}^{R}$—7.8 density (g/cm^3^),
$x_{3}^{R}$—0.27—min value of Poisson’s ratio,
$x_{4}^{R}$—0.3—max value of Poisson’s ratio,
$x_{5}^{R}$—190—min shear modulus (GPa),
$x_{6}^{R}$—210—max shear modulus (GPa),
$x_{7}^{R}$—10.4 × 10^−6^—min thermal expansion coefficient (K^−1^),
$x_{8}^{R}$—10.5 × 10^−6^—max thermal expansion coefficient (K^−1^),
$x_{9}^{R}$—28.6—min thermal conductivity (W/m·K),
$x_{10}^{R}$—28.8—max thermal conductivity (W/m·K).

Digital photo’s parameters:

$x_{1}^{Z}$—2048 vertical resolution (px),
$x_{2}^{Z}$—2048 horizontal resolution (px),
$x_{3}^{Z}$—1—type of color reproduction (e.g., monochrome),
$x_{4}^{Z}$—8—the number of bits mapping colors or shades of gray of a single pixel,
$x_{5}^{Z}$—2—scale (section length in μm mapped by a single bit in the photo).

Inference is carried out using the deductive model [15] based on the bivalent logic. In this solution, the knowledge of the known cases is represented in the form of complex logical rules of evidence and conclusions. The advantage of this is that, assuming that only certain rules will be used (which are reflected in reality), the result will also be devoid of uncertainty. To write the logic rules, Horn clause format was used [16]:

(2) $$ℜ:w_{1}\wedge w_{2}\wedge ...\wedge w_{n}\to k$$

where:

$w_{1}$, $w_{2}$, $w_{n}$—rule evidence; *k*—rule inference.

The next step in the analysis is to identify nitriding areas using the procedures chosen at the application stage.

Formally, this stage is as follows:

$$F_{S2}:X_{S2}\to Y_{S2}$$

where:

$X_{S2}=\{X^{O},X^{P},X^{R}\}$—input values space for the stage concerning the inference associated with the identification of nitriding zones,
$X^{O}=\{x_{1}^{O},x_{2}^{O},...,x_{i}^{O}\}$—set of digital photos,
$X^{P}=\{x_{1}^{P},x_{2}^{P},...,x_{i}^{P}\}$—set of methods of metallographic section preparation parameters,
$X^{R}=\{x_{1}^{R},x_{2}^{R},...,x_{k}^{R}\}$—set of parameters characterizing the core material,
$Y_{S2}=\{Y^{E}\}$—output values space for the stage concerning the inference associated with the identification of nitriding zones,
$Y^{E}=\{y_{1}^{E},y_{2}^{E},...,y_{l}^{E}\}$—set of nitriding zones visualized on the metallographic sections photos.

**Figure 4:**
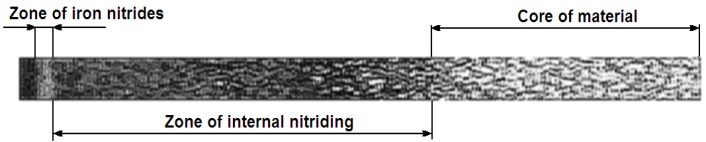
ROI (region of interest) representing the fragment of the nitrided sample metallographic section photo. Tool steel AISI H13.

The analysis is preceded by identifying the ROI (region of interest) [17], which is a part of the image subject to further processing (Figure 4) and contains important information from the point of view of the task.

After determining the ROI, the procedures related to the process of gas nitriding zones' identification, processes associated with improving the image quality are performed, which include, depending on the method of sample preparation and core material parameters:

- Scaling in two-dimensional space,
- Contrast enhancement using one of the functions of the type: 1 − *e*^−*C*^, ln(1 + *C*), tanh(*C*), where: *C*—former contract value,
- Filtration, which aims to compensate the imperfections of the image acquisition process or ineffective exposure of the tested object (filters: median [18]; Gauss [19]).

Later in the process, the identification of nitriding areas is based on an analysis of the brightness distribution of pixels. In this process, the projection of the intensities of pixels on the axis mapping the distance from the surface to the core of the material is carried out by evaluation of the average levels for each row in the ROI starting from the surface of the material according to the equation:

(3) $$\bar{p}(y_{j})=\frac{1}{r_{x}}\sum_{i=1}^{r_{x}}p(x_{i})$$

where:

*x_i_*—number of the next pixel in a single ROI line,
*y_j_*—number of the next pixel in a single ROI row,
*r_x_,r_y_*—the number of pixels in rows and rows forming a rectangular ROI area,
$p(x_{i})$—*i*-th pixel brightness in a ROI row,
$\bar{p}(y_{j})$—the average brightness of the pixels in the j-th row of the ROI area.

**Figure 5:**
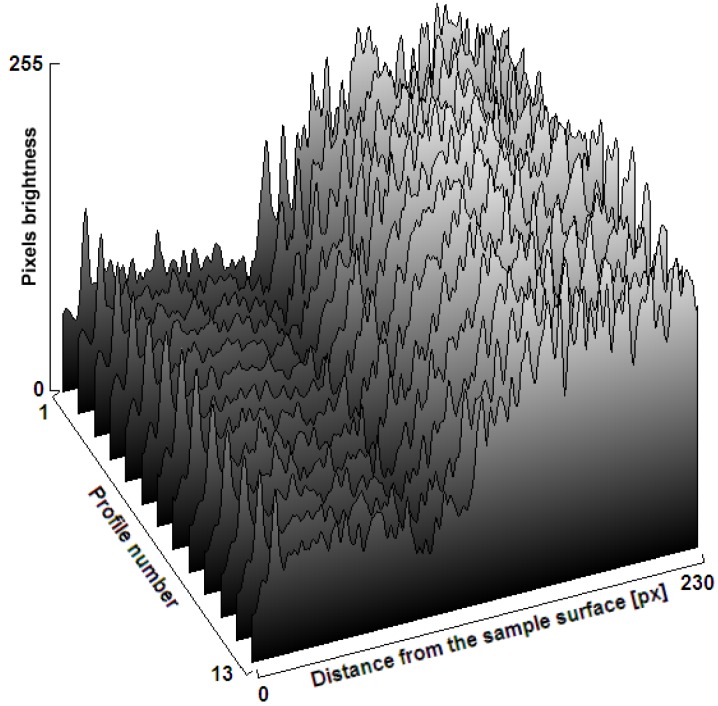
Distribution of the brightness of pixels in an image representing the fragment of the analyzed material (AISI H13 tool steel) from the surface to the core (230 pixels distance) for different profile numbers (pixel rows) in the ROI area.

An example of the pixels’ brightness distribution in an image representing the ROI on the metallographic section of the nitrided sample material is shown in Figure 5.

For the ROI shown in Figure 4, the nitride zone located on the surface of the sample has a higher brightness of pixels with respect to surrounding areas (this zone in many publications is also called a white zone [20]). For this reason, it is possible to detect this in a figure showing the distribution of the brightness of pixels as a peak, whose width also determines the thickness of this zone.

After identifying the location and the thickness of each zone in the sample material pictures, the forecasting process of the hardness distribution in the zone of internal nitriding is being implemented (if this type of zone has been identified).

Formally, this stage is as follows:

$$F_{S3}:X_{S3}\to Y_{S3}$$

where:

$X_{S3}=\{X^{A},X^{R},X^{G}\}$—input values space for predicting the hardness distribution in the zone of internal nitriding,
$X^{A}=\{x_{1}^{A},x_{2}^{A},...,x_{i}^{A}\}$—image data of the analyzed metallographic section,
$X^{R}=\{x_{1}^{R},x_{2}^{R},...,x_{j}^{R}\}$—set of parameters characterizing the core material,
$X^{G}=\{x_{1}^{G},x_{2}^{G},...,x_{k}^{G}\}$—set of parameters characterizing the nitriding zones,
$Y_{S3}=\{Y^{H}\}$—output values space for predicting hardness distribution in the zone of internal nitriding,
$Y^{H}=\{y_{1}^{H},y_{2}^{H},...,y_{l}^{H}\}$—dataset characterizing the projected distribution of hardness in the zone of internal nitriding.

**Figure 6:**
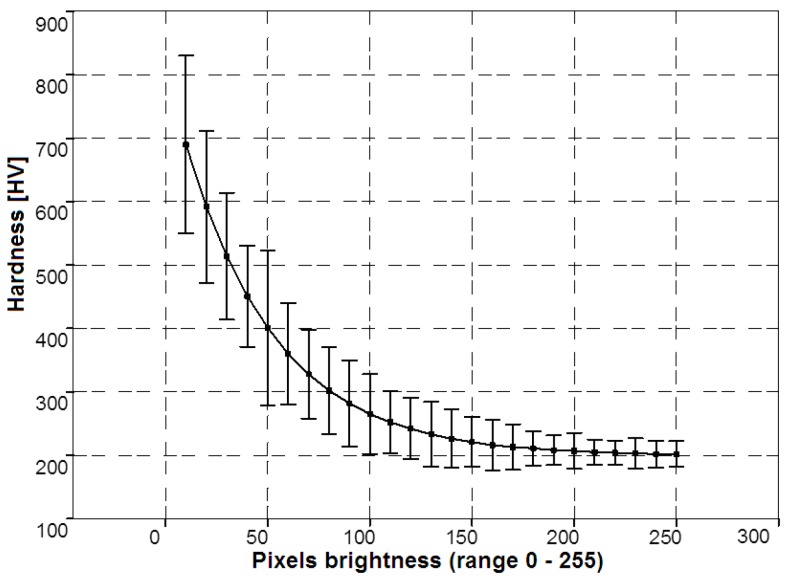
Distribution of the brightness of pixels mapping the hardness of the internal nitriding zone for images of AISI H13 tool steel metallographic sections.

Forecasting the hardness distribution in the zone of the internal nitriding using visual analysis is based on a comparison of the brightness distribution of pixels of the analyzed sample to the pixel brightness distributions of samples made of the same material, for which the characteristics of the hardness distribution are known. Knowledge of these characteristics, *i.e.*, levels of hardness assigned to specific pixel brightness, comes from the hardness tests carried out using the conventional destructive methods carried out on previously-photographed metallographic sections. Hardness is measured for sample points with varying brightness mapped in images. For certain brightness levels multiple measurements of hardness should be made (the average value of hardness is the end result). Research of this type should be done before applying the method developed in the article and should include similar materials as those that are analyzed using the developed method. Metallographic sections of these materials should also be prepared in an analogous manner to the analyzed ones. The larger the collection of empirical data, the higher the chance of finding in the database material similar to that analyzed and subsequently to identify the hardness based on the brightness of pixels. If there is no data available about similar material with a metallographic section prepared in an analogous manner, the hardness estimation is not possible. Hence, the method is heuristic. It was assumed that the average brightness of the pixel intensity values (Figure 6) corresponds to known levels of hardness of the internal nitriding zone.

After determining the distribution of hardness in the internal nitriding zone, another element in the analysis process is carried out: identification of nitriding technology.

Formally, it is as follows:

$$F_{S4}:X_{S4}\to Y_{S4}$$

where:

$X_{S4}=\{X^{W}\}$—input values space for identification of nitriding technology,
$X^{W}=\{x_{1}^{W},x_{2}^{W},...,x_{i}^{W}\}$—set of facts and rules describing the relationship between the parameters entered in the *X^R^* set characterizing the core material, parameters entered in the *X^G^* set characterizing the nitride zones, parameters entered in the *X^H^* set characterizing the predicted hardness distribution,
$Y_{S4}=\{Y^{Z}\}$—output values space for the identification of nitriding technology,
$Y^{Z}=\{y_{1}^{Z},y_{2}^{Z},...,y_{j}^{Z}\}$—collection of adequate gas nitriding technologies allowing one to produce a nitrided layer analogous to the one analyzed.

At this stage, the process is carried out using a similar mechanism as at the stage of the methods of image processing and analysis selection, *i.e.*, using a deductive model and a bivalent logic. The result of inference is a set of potential gas nitriding technologies, using which, it is possible to obtain the outer layers characterized by similar properties to the analyzed sample. The premises and conclusions of rules used at this stage of the analysis include facts describing: nitriding zones (*S*), phases (*F*) in particular zones and gas nitriding technologies (*G*).

where:

$S\in \{S_{a},S_{w}\}$, $F\in \{F_{\varepsilon },F_{\varepsilon +\gamma^{\prime }},F_{\gamma^{\prime }},F_{\alpha +\gamma^{\prime }},F_{\alpha }\}$, $G\in \{G_{1},...G_{n}\}$
$S_{a}\in P(\{F_{\varepsilon },F_{\varepsilon +\gamma^{\prime }},F_{\gamma^{\prime }}\})$,
$S_{w}\in P(\{F_{\alpha +\gamma^{\prime }},F_{\alpha }\})$,
$G_{1},...G_{n}$—sets describing the characteristics of gas nitriding technologies,
*S_a_*—set describing the iron nitrides zone,
*S_w_*—set describing the internal nitrification zone,
$F_{\varepsilon },F_{\varepsilon +\gamma^{\prime }},F_{\gamma^{\prime }}$—sets describing the characteristics of phases in the iron nitrides’ zone,
$F_{\alpha +\gamma^{\prime }},F_{\alpha }$—sets describing the characteristics of phases in internal nitrification zone,
*P*—power set.

Below is an example of a rule for the deductive process of nitriding technology identification:

ℜ: IF $S_{a}=\{F_{\varepsilon +\gamma^{\prime }},F_{\gamma^{\prime }}\}\Rightarrow G=G_{2}$

The knowledge base may include rules describing any gas nitriding technologies, including unregulated or regulated nitriding, with single or multi-component atmospheres, e.g.: NH_3_, NH_3_ + N_2_, NH_3_ + NH_3_dys, NH_3_ + H_2_.

The last step in the process of analysis is to identify the characteristics of the gas nitriding process.

Formally, this stage is as follows:

$$F_{S5}:X_{S5}\to Y_{S5}$$

where:

$X_{S5}=\{X^{C}\}$—input values space for the gas nitriding process characteristics’ identification step,
$X^{C}=\{x_{1}^{C},x_{2}^{C},...,x_{i}^{C}\}$—set of facts and rules describing the relationship between the parameters entered in the *X^R^* set characterizing the core material, parameters entered in the *X^G^* set characterizing the nitride zones, parameters entered in the *X^H^* set characterizing the predicted hardness distribution and identified nitriding technology,
$Y_{S4}=\{Y^{N}\}$—output values space for the gas nitriding process characteristics’ identification step,
$Y^{N}=\{y_{1}^{N},y_{2}^{N},...,y_{j}^{N}\}$—set of parameters constituting the characteristics of the gas nitriding process adequate to produce a nitrided layer analogous to the analyzed one.

In the inference process at this stage, the Mamdani model [21] and fuzzy logic [22] are used. The premises and conclusions of fuzzy rules are constructed with the fuzzy sets mapping the nitriding process parameters. A set of rules to analyze from the set of accumulated rules’ knowledge base is selected based on their conformity with the type of sample core material and the type of technology selected in the previous step. The structure of rules for inference concerning the controlled nitriding technology with a NH_3_ single component atmosphere is as follows:

${ℜ}_{j}$: IF ($F_{\varepsilon }^{0}$ is $M_{j}^{F_{\varepsilon }^{0}}$) AND ($F_{\varepsilon +\gamma^{\prime }}^{0}$ is $M_{j}^{F_{\varepsilon +\gamma^{\prime }}^{0}}$) AND ($F_{\gamma^{\prime }}^{0}$ is $M_{j}^{F_{\gamma^{\prime }}^{0}}$) AND ($F_{\alpha +\gamma^{\prime }}^{0}$ is $M_{j}^{F_{\alpha +\gamma^{\prime }}^{0}}$) AND ($F_{\varepsilon }^{N}$ is $M_{j}^{F_{\varepsilon }^{N}}$) AND ($F_{\varepsilon +\gamma^{\prime }}^{N}$ is $M_{j}^{F_{\varepsilon +\gamma^{\prime }}^{N}}$) AND ($F_{\gamma^{\prime }}^{N}$ is $M_{j}^{F_{\gamma^{\prime }}^{N}}$) AND ($F_{\alpha +\gamma^{\prime }}^{N}$ is $M_{j}^{F_{\alpha +\gamma^{\prime }}^{N}}$) THEN ($Np_{j}$ is $M_{j}^{Np}$) AND ($T_{j}$ is $M_{j}^{T}$) AND ($t_{j}$ is $M_{j}^{t}$)

where:

$F_{\varepsilon }^{0}$, $F_{\varepsilon +\gamma^{\prime }}^{0}$, $F_{\gamma^{\prime }}^{0}$, $F_{\alpha +\gamma^{\prime }}^{0}$—initial widths of particular phases in iron nitrides and internal nitrification zones,
$M_{j}^{F_{\varepsilon }^{0}}$, $M_{j}^{F_{\varepsilon +\gamma^{\prime }}^{0}}$, $M_{j}^{F_{\gamma^{\prime }}^{0}}$, $M_{j}^{F_{\alpha +\gamma^{\prime }}^{0}}$—input space fuzzy sets representing the initial widths of particular phases in iron nitrides and internal nitrification zones,
$F_{\varepsilon }^{N}$, $F_{\varepsilon +\gamma^{\prime }}^{N}$, $F_{\gamma^{\prime }}^{N}$, $F_{\alpha +\gamma^{\prime }}^{N}$—final widths of particular phases in iron nitrides and internal nitrification zones,
$M_{j}^{F_{\varepsilon }^{N}}$, $M_{j}^{F_{\varepsilon +\gamma^{\prime }}^{N}}$, $M_{j}^{F_{\gamma^{\prime }}^{N}}$, $M_{j}^{F_{\alpha +\gamma^{\prime }}^{N}}$—output space fuzzy sets representing the final widths of particular phases in iron nitrides and internal nitrification zones,
$Np_{j}$—nitrogen potential,
*T_j_*—process temperature,
*t_j_*—process duration,
*j* = (1…*m*)—rule number describing the given part of the nitriding process.

The inference at this stage is a complex process with fuzzification (the transformation of sharp signals into fuzzy sets using specific functions that were assumed that will form isosceles triangles with vertices corresponding to the sharp values and the sides stretched throughout the range of the corresponding field), inference (determination of resulting complex fuzzy sets in the output values space for nitrogen potential, temperature and time of the nitriding process) and defuzzification (determination of the sharp values from the complex fuzzy sets using the method of the center of gravity [23]). If the identified nitriding technology includes a multicomponent atmosphere, then the rules are much more complex, because they describe the proportions between the components of the atmosphere, which may change during the nitriding process.

## 4. Model Verification

**Figure 7:**
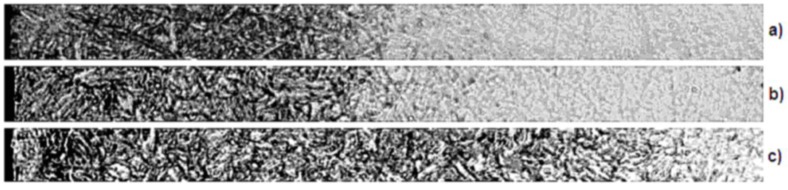
Metallographic section fragments of the gas nitrided steels: (**a**) W300 steel; (**b**) W302 steel; (**c**) W320 steel.

In order to confirm the usefulness of the developed model, verification for the chosen input dataset was performed. These data were images of material samples obtained from gas nitriding processes with varying process parameters. Samples of material before taking pictures were prepared according to established methods (grinding, Nital etching), so that they can provide reference material for the data stored in the system database. There were also collected data on the actual size of the structures represented on digital images of material samples, in order to properly scale them in the image processing and analysis. The database coupled with a knowledge base used by the system contained imaging data, including 22 specimen photos representing fragments of material elements subjected to the gas nitriding processes. Three material samples were chosen for the analysis: W300, W302 and W320 (Figure 7). These steels were chosen due to their widespread use in the machine industry and, thus, demonstrate the usability of the developed methodology.

The verification process takes several steps. The first verification carried out was the process of inference based on the deductive model, concerning automatic selection of analytical procedures consisting of the methods of image processing and analysis.

Of the four developed procedures characterized by certain attributes (the type of core material represented in the pictures, the metallographic section preparation method, the range of pixel brightness in pictures), a procedure was identified, which by the rule knowledge used in the process of inference enables the most effective vision analysis for these cases. This procedure was chosen by means of automatic inference because of the describing rule, joining the sample etching method used and the resulting ranges of variation of pixel brightness.

**Figure 8:**
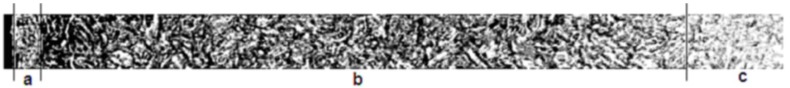
Automatically-identified zones on the picture of the W320 steel metallographic section: (**a**) iron nitrides zone; (**b**) internal nitriding zone; (**c**) material core.

In the next step of verification, the internal nitriding zone and nitrides zones were identified on pictures of material specimens. This was most evident for the W320 steel metallographic section (Figure 8).

**Figure 9:**
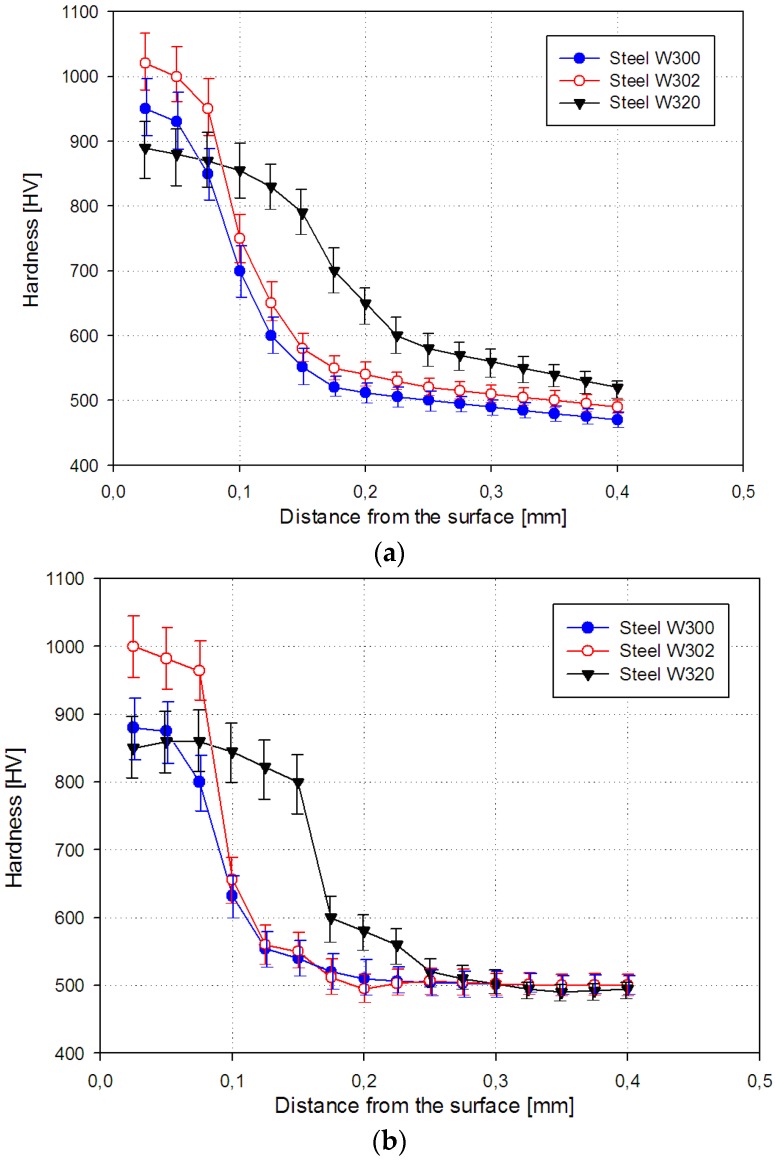
Distribution of hardness for W300, W302 and W320 steels: (**a**) estimated by the developed method based on metallographic section image analysis; (**b**) using the Vickers method.

There were thickness of each zone specified, as well as the hardness in the internal nitriding zone. Hardness estimated using the developed method and Vickers hardness determined for each sample are shown in Figure 9.

Deductive inference-based identification of gas nitriding technologies allow one to obtain material modification corresponding to the analyzed samples, using the indicated potential technologies, including the monocomponent NH_3_ atmosphere, as well as multicomponent NH_3_ + NH_3_zdys, NH_3_ + N_2_. Knowing the sample preparing technology (monocomponent NH_3_ atmosphere-ZeroFlow), the theoretical calculations with the actual parameter values were compared. The values obtained theoretically and the actual parameters used to generate the analyzed material modification are shown in Table 1.

## 5. Conclusions

The developed methodology to identify parameter values determining the nitrided layers’ manufacturing process, as well as material properties, based solely on automated analysis of images of metallographic sections and selected methods of artificial intelligence, provides a solution to the problem of limited access to expensive research equipment. The presented methodology helps also to reduce the human factor in research by automating the process. The methodology should be treated as a heuristic solution due to the need to provide *a priori* knowledge used in the process of automatic inference, which may not cover the required subject range in each analyzed case. In companies of a commercial nature, the developed methodology can be used to discover the methods used to manufacture certain products by the competition. Another area of application can be aiding the analysis of the causes of damage to machines and equipment under field conditions without the need for expensive laboratory equipment and long-term studies. Using only the material specimens’ pictures in analysis is the main limitation of the method, which may in the case of a narrow knowledge base lead to failure to obtain a satisfactory result. One possibility to increase the efficiency of the developed solution is to build an extensive knowledge base to use in the process of inference. Another is to consider the possibility of using other methods, e.g., photoelectron spectroscopy [24], *etc.* The developed model methodology is open, which means that it can be subjected to further modifications, which aim to increase its effectiveness.

## Figures and Tables

**Table 1:** The parameter values calculated for the nitriding process using a monocomponent NH_3_ atmosphere and actual parameter values.

Material Type	W300	W302	W320
The actual parameter values			
Temperature *T* (°C)	570	570	570
Time *t* (h)	8	8	5
Nitrogen potential (Np)	1.5	1.4	6.4
The determined parameter values			
Temperature *T* (°C)	580	580	580
Time *t* (h)	7.5	7.3	4.8
Nitrogen potential (Np)	1.7	1.7	6

Other, less significant parameters, such as retorts and flushing time, were determined independently by the control system of the nitriding device.
